# Anticonvulsant Effects of New 1, 4-DihydropyridineDerivatives Containing Imidazolyl Moiety Against Seizures Induced by Pentylenetetrazole and Maximal Electroshock in Mice

**Published:** 2017

**Authors:** Yasaman Rasouli, Asghar Davood, Armin Alaee, Golnoush Dehqani, Hamed Shafaroudi, Mahboubeh Lotfinia, Mohsen Amini, Abbas Shafiee

**Affiliations:** a *Department of Medicinal Chemistry, Faculty of Pharmacy, Pharmaceutical Sciences Branch, Islamic Azad University, Tehran, Iran. *; b *Department of Pharmacology, Faculty of Pharmacy, Pharmaceutical Sciences Branch, Islamic Azad University, Tehran, Iran. *; c *Department of Medicinal Chemistry, Faculty of Pharmacy and Pharmaceutical Sciences Research Center, Tehran University of Medical Sciences, Tehran, Iran.*

**Keywords:** Dihydropyridine, Imidazole, Anticonvulsant, Pentylenetetrazole, Maximal Electroshock

## Abstract

Epilepsy is a chronic disorder of the brain affecting around 50 million people in the world. Up to 30% of epileptic patients do not respond to available drugs and medical therapies. In this paper, anticonvulsant screening of 10 synthesized new derivatives of 1, 4-dihydropyridine-3, 5-dicarboxamides was performed.

Anticonvulsant activity was evaluated by intravenous and intraperitoneal pentylenetetrazole and maximal electroshock induced seizures tests. Nifedipine was used as reference drug. Our pharmacological results revealing the compounds 2, 4, 5, and 6 can be effective in both absence and grandmal seizures in human.

These pharmacological studies have displayed that these new dihydropyridine derivatives are capable to inhibiting seizures induced by pentylenetetrazole and maximal electroshock in mice efficiently.

## Introduction

Epilepsy is a serious neurological disorder affecting about 1% of the world’s population (~50 million people). It is characterized by spontaneous convulsions and/or a loss of consciousness (1). Many efforts dedicated to the development of novel therapeutics result in promising antiepileptic drugs (AEDs) introduced in the past few decades. However, the currently available anticonvulsant drugs are effective in reducing the severity and number of seizures in less than 80% of patients, up to 30% of patients are resistant to the available medical therapies (2, 3). Despite of using these compounds in a broad range of patients, many of these drugs have dose related side-effects, such as sedation, cognitive impairment, weight gain or birth defects. A few of these compounds may even have rare, but life-threatening side-effects such as serious rash, blood dyscrasias, hepatic failure, or cardiac arrhythmias (4).

Therefore, design and developments of new drugs with lower toxicity, which act by new mechanisms of action, have been strongly advocated. Studies revealed that calcium is an important factor for the induction of epileptic activity. Different seizure-inducing agents or procedures result in to increase the intraneuronal calcium ions concentration, which is thought to be involved in the subsequent epileptiform activity. Also, calcium channel antagonists are effective against a whole range of convulsive procedures, such as electro-convulsions, sound-induced seizures, amygdalar and hippocampal kindling, pentylenetetrazol convulsions, and high pressure-induced seizures (5). Synaptosomal release of excitatory neurotransmitters is dependent on calcium influx, and many established AEDs, such as phenytoin and carbamazepine, inhibit this process by inhibiting calmodulin activation of calmodulin kinase II. Initiation of seizure activity is associated with synchronization of intrinsic burst-firing which is synapsing mediated. The abnormal action potential (paroxysmal depolarizing shift, PDS), which occurs when epileptogenic activity begins, is also dependent on calcium cellular entry (6).

1, 4-dihydropyridines (1, 4 DHPs) are well known as calcium channel blockers and formed a major class of drugs used for the treatment of hypertension. Recently investigations have shown that these compounds indicate variety of biological activities including antimicrobial, myocardial infarction, and neuroprotection activity (7). Also it was proved that the DHPs calcium channel blockers are effective anticonvulsant agents and candidates in experimental seizures (8, 9).

In DHPs replacement of 3, 5-diester moieties with 3, 5-dicarboxamide result in decrease the cardiovascular effect of DHPs (10) so in this study, a series of 3, 5-dicarboxamide-dihydropyridine derivatives were evaluated as anticonvulsant agents against seizures induced by pentylenetetrazole and maximal electroshock in mice.

## Experimental

*Chemistry*

**Scheme 1:**
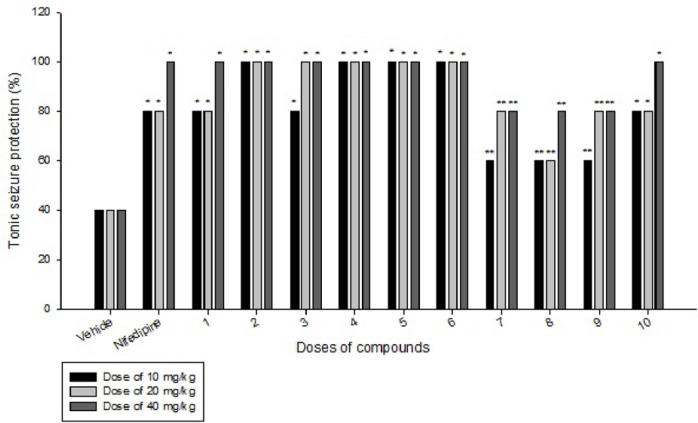
Synthetic procedure of compounds1-10.

Ten new symmetrical dicarboxamide analogues of nifedipine (Table 1) were synthesized according to Scheme 1 (11). The symmetrical analogues were prepared by classical Hantzsch condensation in which 2-methyl (or ethyl)-4(5)-chloro-imidazole-5(4)-carboxal-dehyde (1a–1b) was reacted with 3-oxo-N arylbutanamides and ammonium acetate, and all compounds were really characterized by conventional spectral data. Detail of synthesis and FT-IR, Mass and HNMR of DHPs 1-10 have been reported in our published papers (12, 13).

*Pharmacology*

Male Naval Medical Research Institute (NMRI) mice (20–30 g, Pasteur Institute of Iran) were used in this study. The animals were housed in temperature-controlled room (25 ± 2 °C) on a 12-h light/dark cycle with free access to food and water. They were housed in standard poly carbonate cages and acclimated at least 2 days before experiments. The experiments were performed between 7 and 15 h. All procedures were carried out in accordance with institutional guidelines for animal care and use. Each mouse was used only once, and each treatment group consisted of at least six animals. Pentylenetetrazole (PTZ) was purchased from Sigma (UK). It was dissolved in physiological saline solution. Nifedipine (Tolid Daru, Iran) used as standard compound. All the DHPs derivatives and standard drug were dissolved in dimethylsulfoxide (DMSO) and physiological saline solution.The DMSO/saline vehicle was also used as a control group. Because of toxic properties of DMSO, minimal content of DMSO was used. 

*Determination of clonic and tonic seizure thresholds*

Threshold of PTZ-induced seizures was determined by inserting a 30-gauge butterfly needle into the tail vein of mice and the infusion of PTZ (0.5%) at a constant rate of 1 mL/min to unrestrained animals. Infusion was halted when forelimb clonus followed by full clonus of the body was observed. Minimal dose of PTZ (mg/kg of mice weight) needed to induce clonic seizure was measured as an index of seizure threshold (Equation 1).

*Threshold*= *PTZ* (mg)/ *Weight* (kg)

*Intravenous Pentylenetetrazole induced clonic seizures model*

Dose of 40 mg/kg of the compound 5 was administered intraperitoneally (IP) 0.5% PTZ was administered intravenous (IV) in 30, 60 and 90 min after injection of compound 5, and myoclonic threshold seizure was inspected. Based on time course diagram, the best effect of compound was in 30 and 60 min, efficacy decreased in 90 min. The best selected time was 30 min. Each DHP derivatives was dissolved in DMSO/saline, injected intraperitoneally and screened for anticonvulsant activities at doses of 10, 20 and 40 mg/kg. After 30 min, 0.5% PTZ infused and myoclonic threshold seizure was determined. This procedure repeated for DMSO/saline in order to determine vehicle effect. Also this procedure performed for nifidipine as reference drug at doses of 10, 20 and 40 mg/kg.

**Table 1:** Chemical structure of compounds1-10.

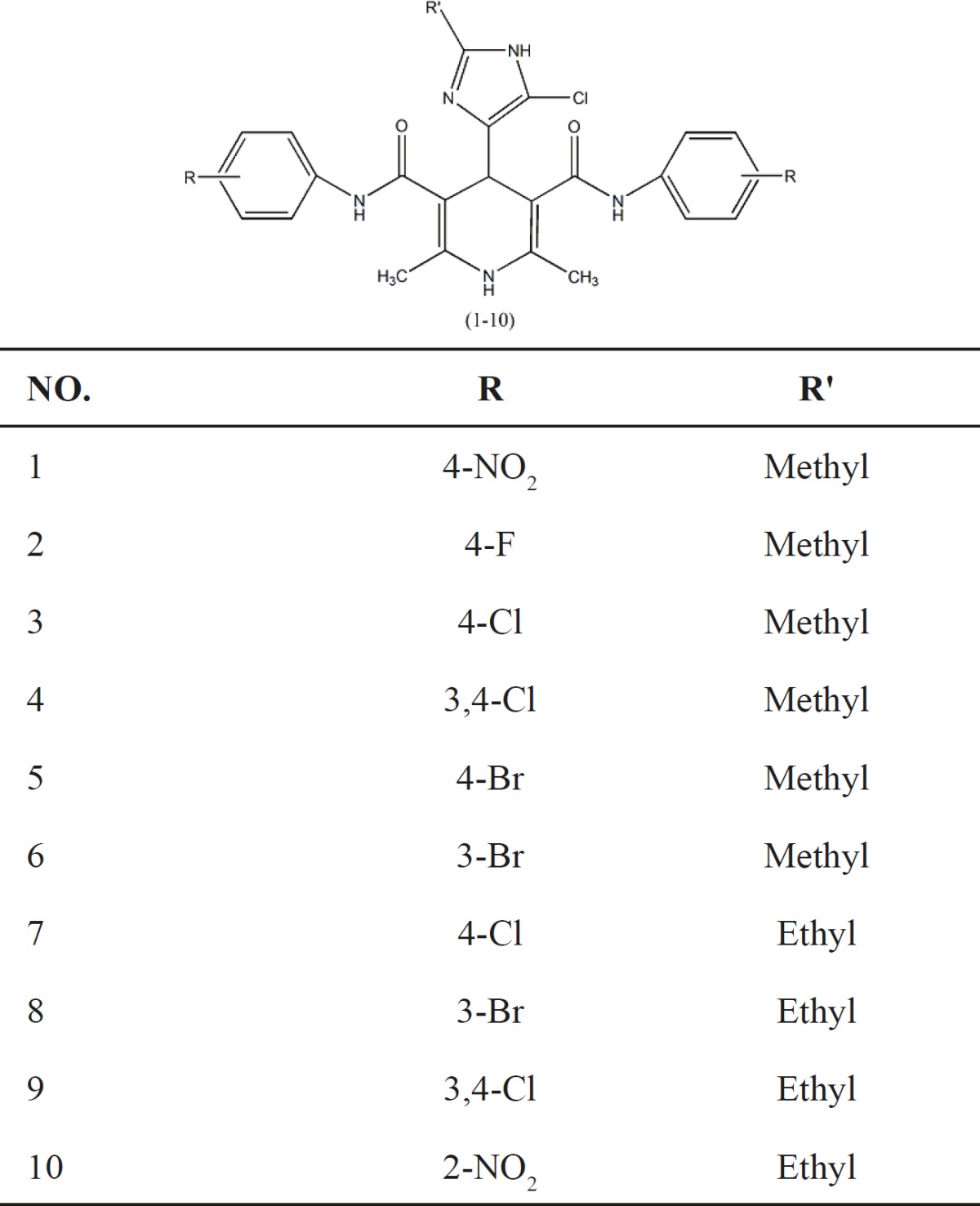

**Table 2:** Effects of IP administration of different doses of compounds (1-10) on IV PTZ-induced seizures

**Comp**.	**CST** a ** in IV PTZ test**			M*w*	Log P
	10 (mg/kg)	20 (mg/kg)	(40 mg/kg)		
1	47.1600±1.9693**5.43×10^-3^ mmol	52.3140±2.4462***0.01 mmol	54.4480±2.4581***0.021 mmol	551.93	4.148
2	59.2360±1.3163***6.02×10^-3^ mmol	60.7280±2.8287***0.012 mmol	61.7440±1.5745***0.024 mmol	497.92	4.686
3	44.9820±3.07575.65×10^-3^ mmol	50.9280±2.3392**0.011 mmol	51.4475±2.7502**0.022 mmol	530.83	5.782
4	49.9120±1.6523***5.00×10^-3^ mmol	54.8960±2.0658***0.01 mmol	60.8200±1.5655***0.02 mmol	599.72	7.026
5	56.4920±1.9882***4.84×10^-3^ mmol	57.4660±0.6922***9.68×10^-3^ mmol	61.0825±2.4022***0.019 mmol	619.73	5.786
6	55.7350±1.3236***4.84×10^-3^ mmol	59.2200±2.5848***9.68×10^-3^ mmol	62.9475±1.5580***0.019 mmol	619.73	5.768
7	37.5525±1.26395.5×10^-3^ mmol	45.7580±3.44730.011 mmol	40.1140±1.94730.022 mmol	544.86	6.035
8	38.1980±2.64314.73×10^-3^ mmol	40.2420±1.86649.46×10^-3^ mmol	40.4940±1.82750.018 mmol	633.76	6.213
9	40.6100±1.13364.88×10^-3^ mmol	43.4975±1.87629.77×10^-3^ mmol	42.8500±2.36630.019 mmol	613.75	27.279
10	44.4325±1.94335.3×10^-3^ mmol	44.7020±1.01770.01 mmol	46.2750±0.9164*0.02 mmol	565.96	4.575
Nifedipine	42.0300±1.03968.66×10^-3^ mmol	47.1200±1.0996**0.017 mmol	54.4920±1.3397***0.034 mmol	346.33	2.20
Vehicle	39.7040±0.8385				

a Clonic seizure threshold, Data are expressed as mean ± SEM

* P < 0.05,

** P < 0.01,

*** P < 0.001 compared to vehicle group.

**Table 3:** Effects of IP administration of different doses of compounds (1-10) on latency of IP PTZ-induced seizures

**Comp.**	**Latency of clonic seizure in IP PTZ test (sec)**			M*w*	Log P
	10 (mg/kg)	20 (mg/kg)	40 (mg/kg)		
1	589.1700±250.1900 5.43×10^-3^ mmol	784.3300±231.0820**0.01 mmol	1268.2000±185.9820***0.021 mmol	551.93	4.148
2	706.3300±155.8360*6.02×10^-3^ mmol	1636.3000±111.0730***0.012 mmol	1725.7000±74.3333***0.024 mmol	497.92	4.686
3	912.5000±238.2180**5.65×10^-3^ mmol	874.3300±301.2010** 0.011 mmol	1303.2000±243.5670***0.022 mmol	530.83	5.782
4	1445.8000±160.8540***5.00×10^-3^ mmol	1723.8000±76.1667***0.01 mmol	1800.0000±0.0000***0.02 mmol	599.72	7.026
5	1415.7000±243.9720***4.84×10^-3^ mmol	1800.0000±0.0000***9.68×10^-3^ mmol	1800.0000±0.0000***0.019 mmol	619.73	5.786
6	1505.0000±189.5240***4.84×10^-3^ mmol	1800.0000±0.0000***9.68×10^-3^ mmol	1800.0000±0.0000***0.019 mmol	619.73	5.768
7	100.000±18.58145.5×10^-3^ mmol	266.3300±45.43180.011 mmol	207.2000±61.51540.022 mmol	544.86	6.035
8	472.6700±117.49904.73×10^-3^ mmol	280.6700±149.33709.46×10^-3^ mmol	247.0000±91.74860.018 mmol	633.76	6.213
9	84.6667±9.81724.88×10^-3^ mmol	416.5000±90.22599.77×10^-3^ mmol	358.8300±59.90460.019	613.75	27.279
10	228.1700±57.94965.3×10^-3^ mmol	382.6700±40.16690.01 mmol	296.3300±93.18210.02 mmol	565.96	4.575
Nifedipine	183.1700±36.76188.66×10^-3^ mmol	870.0000±184.8010**0.017 mmol	1291.7000±2111.6980***0.034 mmol	346.33	2.20
Vehicle	55.6667±7.6667				

Data are expressed as mean ± SEM

* P < 0.05,

** P < 0.01,

*** P < 0.001 compared to vehicle group.

**Table 4:** Effects of IP administration of different doses of compounds (1-10) on frequency of PTZ-induced seizures.

**Compounds**	**frequency of clonic seizure in IP PTZ test (sec)**			M*w*	Log P
	10 (mg/kg)	20 (mg/kg)	40 (mg/kg)		
1	1.8333±0.40145.43×10^-3^ mmol	1.6667±0.333330.01 mmol	1.0000±0.3652*0.021 mmol	551.93	4.148
2	1.5000±0.22366.02×10^-3^ mmol	0.3333±0.2108***0.012 mmol	0.1667±0.1667***0.024 mmol	497.92	4.686
3	1.3333±0.32165.65×10^-3^ mmol	1.1667±0.40140.011 mmol	0.6667±0.3333**0.022 mmol	530.83	5.782
4	0.5000±0.2236***5.00×10^-3^ mmol	0.1667±0.1667***0.01 mmol	0.0000±0.0000***0.02 mmol	599.72	7.026
5	0.3333±0.2108***4.84×10^-3^ mmol	0.0000±0.0000***9.68×10^-3^ mmol	0.0000±0.0000***0.019 mmol	619.73	5.786
6	0.3333±0.2108***4.84×10^-3^ mmol	0.0000±0.0000***9.68×10^-3^ mmol	0.0000±0.0000***0.019 mmol	619.73	5.768
7	1.3333±0.2108*5.5×10^-3^ mmol	1.1667±0.1667**0.011 mmol	1.5000±0.22360.022 mmol	544.86	6.035
8	1.6667±0.21084.73×10^-3^ mmol	2.0000±0.25829.46×10^-3^ mmol	2.0000±0.25820.018 mmol	633.76	6.213
9	1.6667±0.21084.88×10^-3^ mmol	2.1667±0.30739.77×10^-3^ mmol	2.5000±0.56270.019 mmol	613.75	27.279
10	2.5000±0.34165.3×10^-3^ mmol	1.1667±0.1667**0.01 mmol	1.5000±0.22360.02 mmol	565.96	4.575
Nifedipine	1.8333±0.16678.66×10^-3^ mmol	1.1667±0.1667**0.017 mmol	0.6667±0.2108***0.034 mmol	346.33	2.20
Vehicle	2.2000±0.2000				

Data are expressed as mean ± SEM

* P< 0.05,

** P < 0.01,

*** P < 0.001 compared to vehicle group

**Table 5:** Effects of different doses of compounds (1-10) on MES-induced seizures

**Compounds**	**Tonic seizure protection (%)**			p-value*
	10 (mg/kg)	20 (mg/kg)	40 (mg/kg)	
1	80	80	100	P < 0.05
2	100	100	100	P < 0.05
3	80	100	100	P < 0.05
4	100	100	100	P < 0.05
5	100	100	100	P < 0.05
6	100	100	100	P < 0.05
7	60	80	80	P > 0.05
8	60	60	80	P > 0.05
9	60	80	80	P > 0.05
10	80	80	100	P < 0.05
Nifedipine	80	80	100	P < 0.05
Vehicle	40			

* p-values less than 0.05 were considered as indicative of significance.

**Figure 1:**
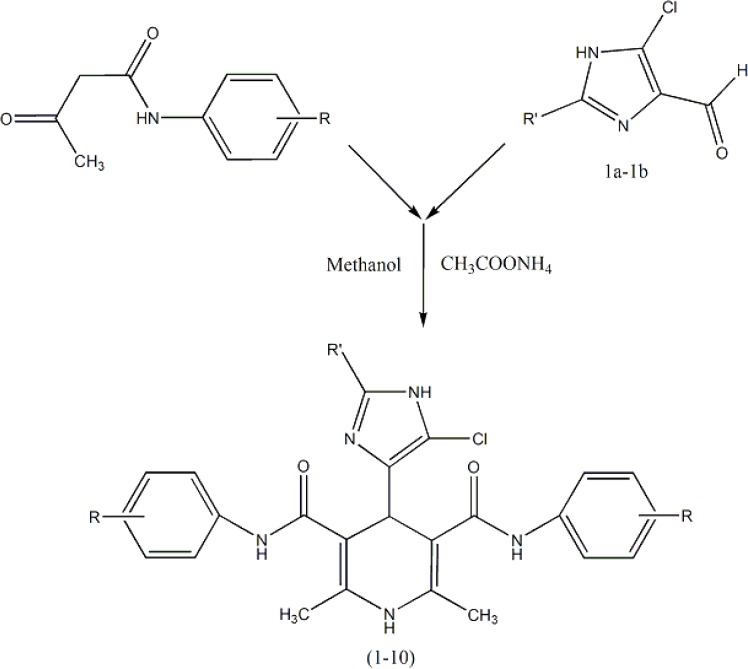
Comparison of IP administration of all compounds at dose of 10 mg/kg, 20 mg/kg and 40 mg/kg on IV PTZ-induced seizure threshold in mice: data are expressed as mean ± SEM of six mice. *P < 0.05, **P < 0.01, ***P < 0.001 compared to vehicle group

**Figure 2:**
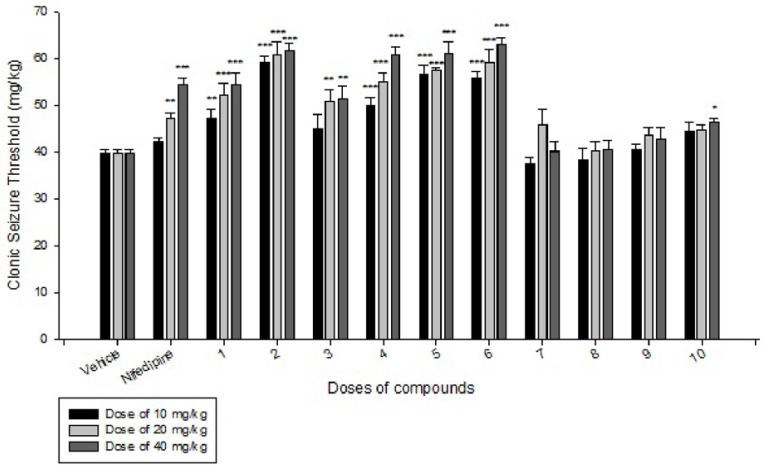
Comparison of IP administration of all compounds at dose of 10 mg/kg, 20 mg/kg and 40 mg/kg on latency of clonic seizure induced by IP PTZ in mice: data are expressed as mean ± SEM of six mice. *P < 0.05, **P < 0.01, ***P < 0.001 compared to vehicle group

**Figure 3:**
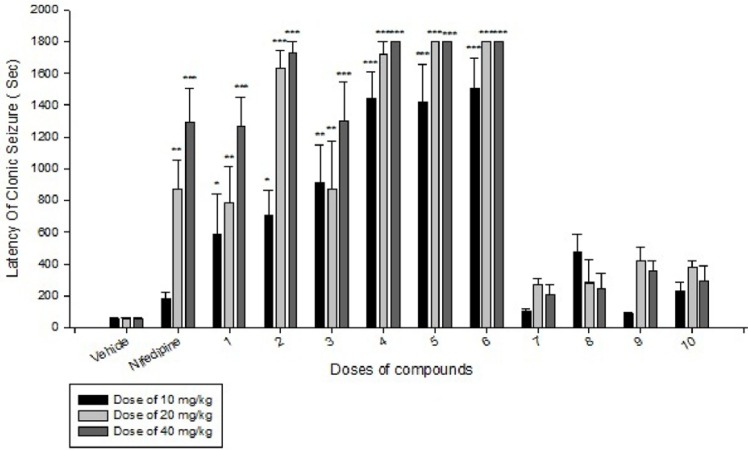
Comparison of IP administration of all compounds at dose of 10 mg/kg, 20 mg/kg and 40 mg/kg on frequency of clonic seizure induced by IP PTZ in mice: data are expressed as mean ± SEM of six mice. *P < 0.05, ***P < 0.001 compared to vehicle group

**Figure 4:**
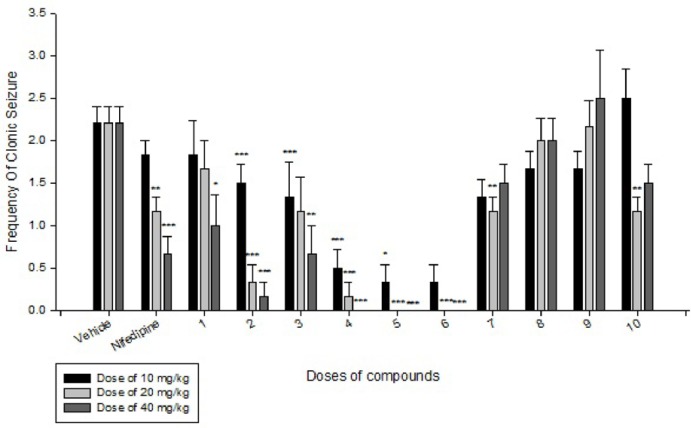
Comparison of IP administration of all compounds at dose of 10 mg/kg, 20 mg/kg and 40 mg/kg on Tonic seizure induced by MES in mice: data are expressed as mean ± SEM of six mice. *P < 0.05, **P > 0.05 compared to vehicle group

*Intraperitoneally Pentylenetetrazole induced clonic seizures model*

Animals were divided to six separated groups and DHP derivatives were injected intraperitoneally at doses of 10, 20 and 40 mg/kg. After 30 min, PTZ was injected intraperitoneally at dose of 85 mg/kg (14). After injection of PTZ, mice were observed for 30 min to detect the seizure latency and duration and frequency and mortality. Seizure latency (SL) is the time that is required to observe the first tonic-clonic-seizure after PTZ injection and seizure duration is the duration (SD) of tonic-clonic seizure. Frequency is the number of seizure attacks in 30 min. This procedure repeated for DMSO/saline in order to determine vehicle effect. Also nifidipine was used as reference drug.

*Maximal electroshock-induced tonic seizures model*

A drop of 0.9% saline was instilled into ears prior to application of electrodes. During the shock, electrodes were attached to each animal’s ears. Maximal electroshock (MES) seizures were elicited with a 20 cycle AC of 35 mA intensity delivered for 0.2 sec via electrodes. Abolition of tonic hind limb extension (THE) component of the seizure was defined as protection in the MES test. Each DHP derivatives was injected intraperitoneally at doses of 10, 20 and 40 mg/kg. After 60 min, MES was performed. Percent of animals that did not involve THE, was calculated.This procedure repeated again for DMSO/saline in order to determine vehicle effect and nifidipine as reference drug.

*Statistical Analysis*

For analyzing data’s, SPSS software (version 20) was used. The results of clonic threshold and latency and frequency of seizures in 30 min are presented as mean ± SEM, and the statistical significance between the groups was analyzed by means of variance followed by one-way ANOVA test and Tukey-krammer test. Analyzing MES data’s was performed by chi-square test. p-values less than 0.05 were considered as indicative of significance.

## Results

Effects of each DHPs derivatives at doses of 10, 20, 40 mg/kg against intravenous pentylenetetrazole-induced seizure on clonic seizure threshold are shown in Table 2. Anticonvulsant effects of the compounds were evaluated by the measurement of seizure latency and frequency via intraperitoneally administration of pentylenetetrazole. Results of latency up to 30 min after administrations of each DHPs derivatives at doses of 10, 20, 40 mg/kg are shown in Table 3. Results of frequency up to 30 min after administrations of each DHPs derivatives at doses of 10, 20, 40 mg/kg are shown in Table 4. Results of MES convulsions at doses of 10, 20, 40 mg/kg are shown in Table 5. Comparison of all compounds against intravenous pentylenetetrazole-induced seizure on clonic seizure threshold at dose of 10, 20 and 40 mg/kg are shown in Figure 1. Comparison of latency up to 30 min after administrations of all compounds at dose of 10, 20 and 40 mg/kg are shown in Figure 2. Comparison of frequency up to 30 min after administrations of all compounds at dose of 10, 20 and 40 mg/kg are shown in Figure 3. Comparison of Tonic seizure protection (%) up to 60 min after administrations of all compounds at dose of 10, 20 and 40 mg/kg are shown in Figure 4. The most potent compounds on clonic seizure threshold are compounds 2, 5, 6. 

The most potent compounds on latency are compounds 4, 5, 6 and the most potent compounds on frequency are compounds 4, 5, 6. The most potent compounds on tonic seizure are compounds 4, 5, 6.

## Discussion

*Clonic seizure*

The anticonvulsant effect of DHPs may be due to the inhibition of L and T type calcium channels and inhibition of N-methyl-D, L-aspartate (NMDLA) receptors and interaction of DHPs with adenosine (12). As shown in the Table 2 and Figure 1, based on the results of IV PTZ-induced seizure (clonic seizure), compounds 1-6 at different doses increased the threshold efficiently more than compounds 7-10, this is may be due to more negative hindrance effect of ethyl group of imidazole moiety in drug-receptor interactions.

In comparison to nifedipine, our designed DHPs derivatives have aryl amide moieties instead of methyl ester group, this replacement result in to increase lipophilicity and consequently better penetration to CNS and better anticonvulsant activity compare to nifedipine. Compound 1 has an electronegative and hydrophilic nitro group in the para position of phenyl ring that result in to participate in hydrogen bonding that can increase the activity more than nifedipine. Compound 2 has an electronegative F group that can participate in hydrogen bond and consequently increase the activity. Comparison of compounds 1 and 2 that contain a strong electronegative group with hydrogen bond ability at position 4 of phenyl ring, reveal that compound 2 is more potent than compound 1, that is due to presence of more hydrophilic nitro group in compound 1 that result in to decrease the lipophilicity and subsequently decrease penetration of compound 1 into CNS. Compound 3 with an electronegative and liphophilic Cl group, is more liphophilic than nifedipine, that difference in logp (partition coefficient) result in to increase the activity of compound 3 in equal doses compare to nifedipine. Due to less electronegativity and π-deficiency ability of Cl to F and NO_2_ group, the compound 3 is less active than compounds 1 and 2. Compound 4 with two electronegative and liphophilic Cl group is more liphophilil and more potent than nifedipine in equal doses. Rationally compound 4 with two Cl groups is more active than compound 3 with one Cl group. Compound 4 which contain 2 electronegative and liphophil Cl group is more active than compound 1 with one strong electronegative but hydrophil group, this shows the importance of logp (partition coefficient) in penetration into CNS and subsequently in anticonvulsant activity. Due to more electronegativity and efficient hydrogen bond ability of F, compound 2 is more active than compound 4. Compounds 5 and 6 which contain more liphophil but less electronegative Br group at 4 and 3 position of phenyl ring respectively, are more active than compounds 1, 3 and 4. Compounds 5 and 6 show less activity than compound 2, because of more electronegative properties and hydrogen bond ability of F group in compound 2. Comparison of compounds 5 and 6 indicate there is no efficient difference between position 4 and 3 of phenyl ring on the anticonvulsant activity in this series of ligands.

Compounds 7, 8, 9 and 10 did not show better anticonvulsant activity than vehicle and their activity is less than nifedipine. Structural difference between compounds 7-10 and compounds 1-6 is in presence of ethyl group instead of methyl group in imidazolyl moiety at position 4 of DHPs ring. Our pharmacological results indicate although ethyl group is more liphophil than methyl group but also it is more bulky than methyl, so its hindrance has negative effect in drug-receptor interaction. The anticonvulsant activity of this series of DHPs show position 4 of DHP ring is very sensitive to bulky substituent and reveals there is a small hydrophobic pocket in receptor to interaction with this part of drugs. Between the compounds 7-10 which contain the ethyl group at position 4 of DHP ring, only compound 10 show slightly anticonvulsant activity that is related to presence of nitro group with hydrogen bond ability at the position 2 of phenyl ring at positions 3 and 5 of DHP ring. Presence of substituent in position 3 or 4 of phenyl ring did not show any difference in activity.

Comparison of all compounds in different doses of 10, 20 and 40 mg/kg reveal except the compounds 7 and 9 (at doses 20 and 40) all the PTZ seizure threshold, increase dose-dependently compared to vehicle group. Based on the results of IP PTZ-induced seizure (clonic seizure), compounds 2, 5, and 6 show the best anticonvulsant activity especially at doses of 10 mg/kg that are more potent than nifedipine at dose of 40 mg/kg.

Based on the results (Table 3 and Figure 2) of IP PTZ-induced seizure (clonic seizure), compounds 1-6 at different doses increased the latency more than compounds 7-10, this is may be due to negative hindrance effect of ethyl group in drug-receptor interactions. At doses of 20 and 40 mg/kg, compounds 2, 4, 5, and 6 show the best effect on latency of clonic seizure than nifedipine.

Based on the results (Table 4 and Figure 3) of IP PTZ-induced seizure (clonic seizure), compounds 1-6 at different doses decreased the frequency more than compounds 7-10, this is may be due to more hindrance effect of ethyl group in drug-receptor interactions. At doses of 20 and 40 mg/kg, compounds 4, 5 and 6 indicate the best effect on frequency of clonic seizure than nifedipine.

Based on the anti-clonic activity of compounds 1-10 it is concluded that the anticonvulsant activity is mainly affected by electronegativity, hydrogen bond ability, and liphophilicity of substituent at phenyl ring in positions 3 and 5 of DHPs ring and hindrance effect of substituent in the position 2 of imidazolyl moiety in the position 4 of DHP ring. 

*Tonic seizure*

Based on the results of MES-induced seizure (tonic seizure, Table 5 and Figures 1-4), compounds 1-6 have equal or more protection effect on tonic seizure than nifedipine. At dose of 10 mg/kg compounds 1 and 3 due to their low liphophilicity, show less effect than other compounds. At dose of 20 mg/kg compound 1 has less effect than other compounds. At dose of 40 mg/kg all of compounds 1-6 show equal effect compared to nifedipine. In compounds 7-10, at dose of 10 mg/kg, only compound 10 due to ability of nitro group to create a hydrogen bond with receptor indicate equal effect compare to nifedipine. At dose of 20 mg/kg compounds 7, 9, and 10 have equal effect compare to nifedipine. At dose of 40 mg/kg only compound 10 has equal effect compare to nifedipine. Based on our pharmacological results it was confirmed that all of our designed and prepared DHPs efficiently protect the tonic seizure. 

## Conclusion

Anticonvulsant screening of 10 synthesized new derivatives of 1,4-dihydropyridine dicarboxamides was performed by intravenous and intraperitoneal pentylenetetrazole and maximal electroshock induced seizures tests. Nifedipine was used as reference drug. Most of dihydropyridine derivatives increased significantly CST induced by IV- PTZ in concentration dependence profile. Also In IP-PTZ, these compounds increase Seizure latency and decrease Frequency in 30 min. Compounds 1-6 at different doses increased the clonic seizure threshold efficiently more than compounds 7-10. This is may be due to unfavorable hindrance effect of ethyl group in compounds 7-10 toward methyl group in compounds 1-6 in drug-receptor interactions. Indeed, this result indicate that position 2 of imidazole moiety is too sensitive to size of substitution and more bulky group like as ethyl result in unfavorable drug-receptor interactions and decrease the potency of these series of DHPs. In comparison to nifedipine as a reference drug, all of compounds 1-6 and compound 10 caused protection against MES tonic seizures at doses of 10, 20, and 40 mg/kg. In general, compounds with the anticonvulsant activity in the petitmal (absence) epilepsy, are effective in PTZ-induced seizure model, compounds which have anticonvulsant activity in MES may be considered as an effective compounds against the grandmal epilepsy (15). Based on our results, compounds 2, 4, 5 and 6 may be effective in both absence and grandmal seizures in human. 

As have shown already, in DHPs replacement of 3, 5-diester moieties with 3,5-dicarboxamide results in to decrease the cardiovascular effect of DHPs (10). Based on results of our research, this replacement increases anticonvulsant activity more than nifedipine. So, design of new 1, 4-dihydropyridine-3, 5-dicarboxamide derivatives with different hydrophilic or lipophilic groups, can achieve compounds with better anticonvulsant effects and less adverse drug reactions.
